# Association of Procalcitonin with the Patient's Infection Characteristics and Prognosis after Hematopoietic Stem Cell Transplantation

**DOI:** 10.1155/2022/9157396

**Published:** 2022-09-13

**Authors:** Shan-Shan Li, Jun-xu Gu, Xiao-wei Li, Na Zhang, Mei Jia, Lin Pei, Ming Su

**Affiliations:** ^1^Department of Clinical Laboratory, Peking University People's Hospital, Beijing, China; ^2^Department of General Surgery, Beijing Rehabilitation Hospital of Capital Medical University, Beijing 100144, China

## Abstract

**Objective:**

To study whether procalcitonin (PCT) is an important indicator of infection with or without agranulocytosis and to reveal whether PCT can distinguish between infected sites and affect prognosis after hematopoietic stem cell transplantation (HSCT).

**Method:**

In the present study, 682 patients with HSCT were enrolled, and their clinical characteristics were noted. Their blood culture and inflammatory and biochemical indicators were studied. The patients were divided into respective groups according to the degree of agranulocytosis, type of bacterial infection, infected sites, and prognosis.

**Results:**

The PCT, CRP, and D-dimer levels were significantly improved in patients with positive blood culture results compared to the case for those with negative blood culture results. The PCT level was the highest in the gram-negative group. The levels of PCT and D-dimer were significantly elevated in patients with infection and agranulocytosis after HSCT compared to those in the nonagranulocytosis cohort. Interestingly, no significant difference in the PCT level was observed among any of the eight foci. Lower PCT levels were associated with higher survival in patients with infection after HSCT.

**Conclusion:**

Among patients that underwent HSCT, PCT levels were significantly elevated in those with infection and agranulocytosis, with the levels being specifically high in the gram-negative group. Moreover, lower PCT levels were associated with higher survival in patients with infection after HSCT.

## 1. Introduction

Patients that undergo hematopoietic stem cell transplantation (HSCT) face a high risk of bacteremia soon after HSCT because of major insults to their innate immune system. These patients experience prolonged neutropenia upon receiving their conditioning regimen and, therefore, lack the primary phagocytes that are essential for combating bacterial infections [1]. In addition, the conditioning regimen results in marked gastrointestinal mucositis, which damages the integrity of their mucosal barrier [2]. These two key insults establish a high-risk setting for infections, especially, bacteremia caused by enteric organisms.

Infectious complications remain a major issue in patients after HSCT. The most common manifestation of infection is fever; however, various types of noninfectious fever can also develop. Distinguishing between infectious conditions and aseptic causes of febrile events is difficult in patients with HSCT because of transplant-related complications, such as graft-versus-host disease, engraftment syndrome, thrombotic microangiopathy, and relapse of underlying diseases [3]. Early distinction of infection-related fever and immediate antibiotic treatment are extremely important for treating these patients. Therefore, adequate and timely clinical decision-making is important, and a blood culture is recommended for patients with suspected systemic bacterial infection [4–6].

Blood culturing, being a slow process, delays the results; therefore, clinicians need specific indicators to guide the early use of appropriate antibiotics at an early stage to treat infections. Popular biomarkers of infections include C-reactive protein (CRP) and procalcitonin (PCT). The PCT concentration in the blood is elevated by systemic infections, especially, bacterial infections that cause severe illness, but not by local infection. The PCT levels can be utilized to diagnose different infectious diseases caused by bacteria [7] and can discriminate between different etiologies of infection, namely, gram-positive cocci, gram-negative bacilli, and fungi [8]. Whether PCT can differentiate between bacterial infections from fevers with other etiologies in patients with HSCT remains controversial. Further, whether PCT is associated with the site of infection and can predict disease outcome in patients with HSCT remains unclear.

The objective of this study was to analyze HSCT patients with fever and investigate the potential of the PCT and CRP levels in predicting and diagnosing systemic bacterial infection and the possibility of future infections.

## 2. Materials and Methods

### 2.1. Patients

A total of 4 404 hematology in-patients undergoing chemotherapy or HSCT in the Peking University People's Hospital between October 2016 and May 2020 participated in this study. A total of 682 patients were enrolled in this study after applying the exclusion criteria (Figure S1). Among them, 197 had contracted infections after HSCT, and their blood culture results were positive; 485 patients had negative blood culture results (Table S1). The study protocol was approved by the Hospital Research Ethics Committee, and written consent was obtained from all patients.

### 2.2. Methods

All test samples (including PCT and blood culture) were collected within the first 12 h after the development of fever in patients. The “criterion of fever” was a body temperature ≥ 38.0°C [9]. Blood samples were cultured using an American BD FX400 automatic blood culture system (Becton, Dickinson and Company, USA). Blood culture results were defined as negative (no infection) if there was no microbial growth after five days. The PCT levels were measured using a Roche E411 autoimmune analyzer (Roche Diagnostics, Germany), and D-dimer levels were analyzed using an ACL TOP700 analyzer (Werfen Inc., Spain); the serum biochemical indices were measured using an AU5832 automatic biochemical analyzer (Beckman Coulter Inc.). The blood routine index was analyzed using an XN-20 analyzer (SYSMEX, Japan).

### 2.3. Statistical Analyses

Data were analyzed using the SPSS 25 software (IBM, Armonk, USA). The discrete variables were presented as percentages (%), and the continuous variables were presented as the means with standard deviations (SDs) or median with interquartile range (IQR) between the 25th and 75th percentiles. For nonparametric data, two datasets were compared using the Mann–Whitney *U* test, and three or more datasets were compared using the Kruskal–Wallis *H* test. We generated Kaplan–Meier survival curves, for which the observation period was defined as 70 days from blood sample collection for blood culture and the event was defined as death occurring during these 70 days. The threshold for significance was set at *p* < 0.05.

## 3. Results

### 3.1. PCT Level Was Significantly Elevated in Patients Showing Positive Blood Culture Results with Agranulocytosis and Was Particularly High in the Gram-Negative Group after HSCT

The levels of PCT, CRP, and D-dimer were significantly elevated in patients with positive blood culture results (patients with infection) compared to those in the patients without infection (Table S1). Among the patients with infection, 76, 113, and 8 patients showed infections with gram-positive bacteria, gram-negative bacteria, and fungi, respectively. The PCT levels and WBC counts were much higher in patients with bacteremia caused by gram-negative pathogens than in those with bacteremia caused by gram-positive pathogens and fungal infections (*p* < 0.05; Table S1). The 197 patients with infections were divided into two groups according to the degree of agranulocytosis.  Table 1 shows the distribution of hematologic diseases in the patients. The median age of the patients from the agranulocytosis group, which comprised 108 (90.8%) men, was 40 (26–51) years. The median age of the patients from the nonagranulocytosis group, which comprised 67 (85.9%) men, was 36 (26–50) years. The PCT levels in the agranulocytosis group (1.64 (0.25–10.12) ng/mL) were much higher than those in the nonagranulocytosis group (0.76 (0.28–2.72) ng/mL) (*p* < 0.005). The D-dimer levels in the agranulocytosis group (455.00 (272.00–868.00) mg/mL) were significantly lower than those in the nonagranulocytosis group (918.50 (445.50–3716.00) mg/mL) (*p* < 0.005). The activities of lactate dehydrogenase (LDH), alanine transaminase (ALT), and alkaline phosphatase (ALP) in the agranulocytosis group were significantly higher than those in the nonagranulocytosis group (Table 1). No significant differences between the levels of CRP, albumin (ALB), blood glucose (GLU), creatinine (CRE), blood urea nitrogen (BUN), and brain natriuretic peptide (BNP) and the activities of aspartate transaminase (AST) and creatine kinase (CK) were noticed between the two groups (Table 1).

We further grouped the patients and found that among patients from the agranulocytosis groups, the PCT levels in patients with infections caused by gram-negative bacteria (2.00 (0.40–8.35) ng/mL) were significantly higher than those in patients with infections caused by gram-positive bacteria (0.35 (0.20–1.95) ng/mL). Similarly, in the nonagranulocytosis groups, the PCT levels in patients with infections caused by gram-negative bacteria (0.98 (0.30–6.78) ng/mL) were significantly higher than those in patients with infections caused by gram-positive bacteria (0.31 (0.16–0.90) ng/mL) (Table 2).

### 3.2. PCT Levels Were Not Associated with the Definite Focus of Infection

The PCT levels in bacteremia patients with eight different foci of infection, of which pulmonary and perianal infections were most common, are shown in Table S2. No significant differences in PCT levels were noticed among the eight foci. The PCT levels in patients with infections caused by gram-positive and gram-negative bacteria were also compared after grouping these patients according to the presence or absence of a definite focus of infection. Among patients with and without a definite focus on infection, the PCT levels in patients with infections caused by gram-negative bacteria were significantly higher than those in patients with infections caused by gram-positive bacteria (*p* < 0.05 for both; Table 3). The ALB levels in patients without a definite focus of infection (36.10 (32.00, 37.45) g/L) were significantly higher than those in patients with a definite focus of infection (32.95 (30.53, 37.45) g/L) (*p* < 0.05). No significant differences were noticed between the levels of other parameters among the patients from the different groups (Table S3).

### 3.3. Lower PCT Values Were Associated with Higher Survival in Patients after HSCT

**Figure 1:**
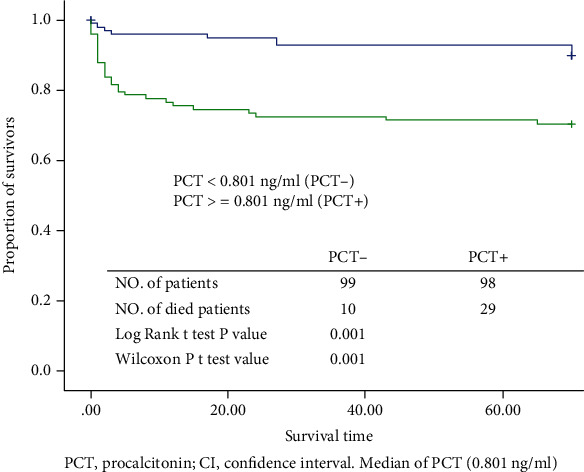
Kaplan–Meier survival plot showing proportion of survivors with a risk of bacterial infection. PCT: procalcitonin; CI: confidence interval. Median of PCT (0.801 ng/mL).

The patients were divided into two groups on the basis of their condition during discharge, i.e., whether their condition showed improvement (improvement group) or whether they died (death group). The PCT levels; WBC counts; D-dimer levels; ALT, LDH, and CK activities; and CRE, BUN, BNP, and GLU levels were significantly higher in patients who died than in those whose condition showed improvements (*p* < 0.005 for all). However, the CRP and ALB levels were lower in patients who died than in those whose condition showed improvements (*p* < 0.005 for both; Table 4). Depending on the median PCT level (cut-off value, 0.801 ng/mL), the patients were divided into two groups: PCT-negative (PCT-), <0.801 ng/mL, and PCT-positive (PCT+), ≥0.801 ng/mL. The results of the survival analysis of patients from the PCT+ and PCT- groups are shown in Figure 1. The survival rate was much lower in the PCT+ group than in the PCT- group (log-rank test, *p* = 0.001).

## 4. Discussion

Procalcitonin levels are clinically significant in patients with neutropenia or undergoing HSCT [10, 11]. Our study showed that the levels of PCT and D-dimer were more accurate than CRP levels in discriminating between bacteremia and nonbacteremia in patients with infectious diseases and agranulocytosis after HSCT and that PCT testing could distinguish between infections caused by gram-negative and gram-positive bacteria. These findings are in agreement with the results of a study that reported that PCT was more reliable than CRP for discriminating between bacteremia and nonbacteremia, independent of neutropenia or HSCT [12]. Moreover, PCT has an important clinical role in febrile neutropenia. Further issues, such as the validation of a specific decision algorithm that includes PCT to monitor the choice of antibiotic and determine the treatment duration, can be addressed in prospective studies [13]. PCT might provide additional information and could be used in combination with other biomarkers to detect infections in patients with hematological malignancies [14]. Moreover, the combination of PCT, interleukin- (IL-) 6, and D-dimer analyses enhances the diagnostic capability for sepsis and severe sepsis [15]. Combined measurement of PCT with Alb is expected to be a valuable tool to assess prognosis in elderly people at risk of bacterial infection [16]. High D-dimer levels have been associated with 28-day mortality in patients with infection or sepsis [17]. High serum IL-8 and D-dimer levels can be useful markers to identify patients with chemotherapy-induced neutropenia [18]. However, PCT may be the only marker to distinguish between infections caused by gram-negative and gram-positive bacteria, as the levels of CRP and D-dimer are not reliable markers to distinguish between these infections.

The PCT levels are related not only to gram-positive or gram-negative bacteria but also specific pathogens and, particularly, the infection sites. Further, PCT levels are an important factor in determining the use of antibiotics [19, 20]. Our study showed that PCT levels are not associated with the definite focus of infection. However, PCT could distinguish between infections caused by gram-negative and gram-positive pathogens at each site of infection after HSCT. However, these results have some limitations. PCT levels have been reported to distinguish between infections by gram-negative and gram-positive bacilli, as well as between different bacterial species and infection sites [21]. This could be explained by the fact that the site of colonization differs among pathogens. Some infection sites are dominated by gram-negative bacteria, while others are dominated by gram-positive bacteria or have a similar bacterial composition.

Our study showed that PCT levels were significantly higher in patients who died after HSCT, and the survival rate was significantly lower in the PCT+ group than in the PCT- group. These results are consistent with those from other previous studies. For HSCT patients with serious conditions, an elevated PCT level has been associated with an increased risk of mortality (RR 4.18, 95% CI: 3.19–5.48) [22]. Early measurement of serum PCT levels may be useful in predicting mortality in patients with hematological malignancy who require advanced life support [23, 24]. A previous study conducted in China reported that PCT might serve as a predictive indicator of post-HSCT 100-day mortality, where a nomogram constructed based on the PCT level and several clinical factors can effectively predict the 100-day survival of febrile patients [25].

Inflammatory factors, such as neutrophil lymphocyte ratio (NLR), CRP, and PCT, have important clinical applications in assessing the extent of disease and prognosis of patients with bloodstream infection and sepsis [26–28]. Dynamic monitoring of serum PCT levels can help assess the prognosis of septic shock patients and predict the severity of illness; however, it may not be a significantly independent prognostic marker to assess survival in patients with sepsis [29].

This study had some limitations. First, PCT levels can vary with infection time, specifically during the first 6 h of infection [30, 31]. The ability of PCT to distinguish between infections caused by gram-negative and gram-positive bacteria may have been weakened by the fact that the intervals between the symptoms and sampling times varied. Second, since this was a single-center, single-department study and included only patients who experienced severe infection after HSCT, these observations cannot be generalized to the general population of patients with infections. Finally, this study used a small sample size, and multicenter studies are warranted to validate these experimental observations.

In conclusion, PCT was significantly elevated in HSCT patients showing positive blood culture with agranulocytosis and was particularly higher in patients with infections caused by gram-negative pathogens. Moreover, lower PCT levels were associated with higher survival in patients showing positive blood culture results after HSCT.

## Figures and Tables

**Table 1:** Patients with positive blood culture results.

	Number of all	Agranulocytosis	No agranulocytosis	*p*
Number of all	197 (100%)	119 (60%)	78 (40%)	—
Number of ALL	65 (33%)	36	29	—
Number of AML	81 (41%)	51	30	—
Number of CML	9 (4.5%)	5	4	—
Number of MDS	25 (13%)	16	9	—
Number of lymphoma	6 (3%)	5	1	—
Number of AA	9 (4.5%)	5	4	—
Number of MF	1 (0.5%)	1	0	—
Number of MM	1 (0.5%)	0	1	—
PCT (ng/mL)		1.64 (0.25, 10.12)	0.76 (0.28, 2.72)	<0.05
CRP (mg/L)		85.73 (52.06, 144.80)	54.84 (17.01, 114.55)	0.281
D-Dimer (mg/mL)		455.00 (272.00, 868.00)	918.50 (446.00, 3716.00)	<0.05
AST (U/L)		32.50 (21.00, 56.25)	48.00 (28.50, 70.00)	0.615
ALT (U/L)		23.00 (16.00, 33.75)	44.50 (24.00, 63.50)	<0.05
LDH (U/L)		157.50 (128.00, 206.75)	397.50 (264.50, 704.75)	<0.05
ALP (U/L)		63.50 (46.50, 78.00)	111.50 (60.5, 208.00)	<0.05
CK (U/L)		21.50 (17.25, 36.00)	33.00 (14.25, 73.25)	0.139
ALB (g/L)		34.05 (31.60, 37.05)	30.80 (28.63, 37.97)	0.560
CRE (*μ*mol/L)		61.00 (47.25, 85.00)	55.00 (42.00–103.50)	0.795
BUN (mmol/L)		5.56 (4.15, 8.82)	6.51 (4.30, 14.97)	0.221
GLU (mmol/L)		6.61 (5.48, 6.47)	6.31 (4.99, 8.44)	0.083
BNP (p g/mL)		155.00 (49.50, 595.10)	223.05 (90.00, 1090.00)	0.432
Hb (g/L)		81.00 (74.50, 89.00)	84.00 (66.75, 101.00)	0.568
Age (years)		40 (26, 51)	36 (26–50)	<0.05
Body temperature		38.70 (37.82, 39.20)	38.55 (37.43–39.00)	0.203
Sex (male%)		108 (90.8%)	67 (85.9%)	—

Data are presented as the median value and interquartile range (IQR). ALL: acute lymphoblastic leukemia; AML: acute myeloid leukemia; CML: chronic myelocytic leukemia; MDS: myelodysplastic syndrome; MM: multiple myeloma; MF: myelofibrosis; AA: aplastic anemia; CRP: C-reactive protein; PCT: procalcitonin; AST: aspartate transaminase; ALT: alanine transaminase; LDH: lactate dehydrogenase; ALP: alkaline phosphatase; CK: creatine kinase; BUN: blood urea nitrogen; BNP: brain natriuretic peptide; Hb: hemoglobin; ALB: albumin; CRE: creatinine; GLU: blood glucose.

**Table 2:** PCT levels in patients with bacteremia infection according to agranulocytosis after HSCT (based on gram staining results).

	Microorganisms	Patients (*n*; %)	PCT (ng/mL) median (IQR)	PCT (ng/mL) mean	*p*
Agranulocytosis	Gram-negative	83 (44%)	2.00 (0.40, 8.35)	13.58	0.002
	Gram-positive	31 (16%)	0.35 (0.20, 1.95)	5.11	
No agranulocytosis	Gram-negative	30 (16%)	0.98 (0.30, 6.78)	8.02	0.002
	Gram-positive	45 (24%)	0.31 (0.16, 0.90)	2.00	

**Table 3:** PCT levels in patients with bacteremia infection according to infectious focus after HSCT (based on gram staining results).

	Microorganisms	Patients (*n*; %)	PCT (ng/mL) median (IQR)	PCT (ng/mL) mean	*p*
Patients without definite focus of infection	Gram-negative	47 (25%)	1.98 (0.34, 10.43)	10.55	0.001
	Gram-positive	34 (18%)	0.31 (0.14–0.91)	4.26	
Patients with definite focus of infection	Gram-negative	66 (35%)	0.92 (0.40, 6.67)	13.21	0.002
	Gram-positive	42 (22%)	0.32 (0.20, 1.99)	2.46	

**Table 4:** Comparison of inflammatory and biochemical indicators between patients whose condition showed improvement and those who died.

	Improvement (*n* : % = 158 : 80%)	Died (*n* : % = 39 : 20%)	*p*
PCT (ng/mL)	0.66 (0.24, 6.94)	2.16 (0.76, 23.84)	<0.05
CRP (mg/L)	82.66 (35.52, 123.81)	69.39 (40.88, 172.75)	<0.05
WBC (10^9^ cells/L)	0.01 (0.00, 0.31)	0.39 (0.02, 10.31)	<0.05
Hb (g/L)	81.50 (76.00, 89.00)	92.00 (68.25, 101.00)	0.265
D-Dimer (mg/mL)	449.00 (261.25, 800.00)	1841.00 (524.00, 3907.50)	<0.05
AST (U/L)	38.00 (23.75, 55.50)	41.50 (23.75, 80.75)	0.431
ALT (U/L)	23.00 (16.75, 40.00)	41.00 (24.66, 75.00)	<0.05
LDH (U/L)	163.00 (128.00, 218.75)	394.50 (235.00, 734.00)	<0.05
ALP (U/L)	66.00 (50.75, 92.50)	67.00 (47.50, 208.00)	0.064
CK (U/L)	20.50 (15.30, 25.00)	59.50 (25.75, 220.00)	<0.05
ALB (g/L)	34.25 (31.60, 37.20)	29.30 (26.53, 35.43)	<0.05
CRE (*μ*mol/L)	57.50 (44.00, 75.50)	82.00 (52.50, 110.75)	<0.05
Age (years)	41.50 (26.00, 52.00)	34.00 (25.00, 48.25)	0.960
Body temperature	38.70 (37.90, 39.00)	38.45 (37.08, 39.20)	0.480
BUN (mmol/L)	5.06 (3.58, 6.92)	14.58 (6.35, 16.72)	<0.05
BNP (p g/mL)	102.00 (37.13, 406.13)	844.25 (287.50, 1612.50)	<0.05
GLU (mmol/L)	6.36 (5.06, 8.28)	7.35 (5.15, 9.76)	<0.05
Agranulocytosis (%)	100 (159) 62.9%	19 (39) 48.7%	—

## Data Availability

The authors can be reached by email.

## Supplementary Materials

Supplementary Materials Table S1: comparison of PCT, WBC, CRP, D-dimer levels in all the patients. Table S2: PCT values in infection patients with different focus of infection. Table S3: comparison of a variety of inflammatory and biochemical indicators between different focus of infection. Figure S1: flow chart of selection of 682 patients.
