# Supplementary material for: Association of Procalcitonin with the Patient's Infection Characteristics and Prognosis after Hematopoietic Stem Cell Transplantation
Source: Dis Markers. 2022 Sep 13;2022:9157396. doi: 10.1155/2022/9157396 (PMC9489411; doi:10.1155/2022/9157396)
Supplement: Supplementary Materials — Table S1: comparison of PCT, WBC, CRP, D-dimer levels in all the patients. Table S2: PCT values in infection patients with different focus of infection. Table S3: comparison of a variety of inflammatory and biochemical indicators between different focus of infection. Figure S1: flow chart of selection of 682 patients. [file 9157396.f1.docx]

Table S1 Comparison of PCT, WBC, CRP, D-Dimer levels in all the patients.

|  | Negative blood culture  (n; %=485;71%) | Positive blood culture  (n; %=197;29%) | *p* | Gram-positive  (n; %=76;39%) | Gram-negative  (n; %=113;57%) | Fungus  (n; %=8;4%) | *P* |
| --- | --- | --- | --- | --- | --- | --- | --- |
| PCT  (ng/ml) | 0.19  (0.12,0.30) | 1.98  (0.56,6.07) | <0.05 | 0.31  (0.19,1.32) | 1.96  (0.38,7.92) | 0.87(0.15,1.69) | <0.05 |
| CRP  (mg/L) | 41.02  (13.37,94.10) | 68.21  (23.60,123.15) | <0.05 | 68.06  (29.77,116.51) | 68.78  (23.81,136.64) | 69.16  (14.29,123.37) | 0.881 |
| WBC  (10^9 cells/L) | 1.45  (0.00,5.71) | 0.30  (0.00,0.72) | <0.05 | 1.35  (0.00,5.30) | 0.02  (0.00,0.68) | 0.06(0-4.77) | <0.05 |
| D-Dimer  (mg/ml) | 335.00  (183.00,716.00) | 439.00  (244.00,874.00) | <0.05 | 453.00  (228.00,1000.00) | 435.00  (222.00,953.00) | 565.00  (313.00-904.00) | 0.652 |

CRP: C-reactive protein; PCT: procalcitonin;

Table S2 PCT values in infection patients with different focus of infection

|  | n; % | PCT (ng/mL) | *P* |
| --- | --- | --- | --- |
| Patients without definite focus of infection | 85(43%) | 0.77（0.23，5.48） | 0.309 |
| Lung | 26(13%) | 0.44（0.22，2.37） |  |
| Perianal region | 25(12%) | 1.89（0.24，5.69） |  |
| Digestive system | 19(10%) | 2.70（0.36，7.39） |  |
| Mouth cavity | 15(8%) | 0.42（0.20，2.60） |  |
| Catheter | 11(6%) | 1.01（0.30，2.09） |  |
| Urinary system | 8(4%) | 0.31（0.22，40.44） |  |
| Skin | 6(3%) | 2.60（0.37，12.79） |  |
| Knee | 2(1%) | (0.70，0.08) |  |

Table S3 Comparison of A variety of inflammatory and biochemical indicators

between different focus of infection.

|  | Patients without definite focus of infection (n; %=85:43%) | Patients with definite focus of infection (n: %=112:57%) | *P* |
| --- | --- | --- | --- |
| ALB (g/L) | 36.10(32.00,37.45) | 32.95(30.53,37.45) | <0.05 |
| PCT (ng/ml) | 0.78(0.24,5.22) | 0.86(0.27,3.96) | 0.66 |
| CRP (mg/L) | 73.92(23.97,133.60) | 67.18(24.86,124.21) | 0.921 |
| WBC (10^9 cells/L) | 0.05(0.00,2.53) | 0.10(0.00,2.30) | 0.984 |
| D-Dimer(mg/ml) | 437.00(214.00,934.00) | 449.00(228.00,987.00) | 0.475 |

Figure S1 Flow chart of selection of 682 patients.
